# Propagule pressure increase and phylogenetic diversity decrease community’s susceptibility to invasion

**DOI:** 10.1186/s12898-017-0126-z

**Published:** 2017-04-11

**Authors:** T. Ketola, K. Saarinen, L. Lindström

**Affiliations:** Department of Biological and Environmental Science, Centre of Excellence in Biological Interactions, University of Jyvaskyla, P.O. Box 35, 40014 Jyvaskyla, Finland

**Keywords:** Bacteria, Competition, Invasion, Phylogenetic distance, Phylogenetic similarity and propagule pressure

## Abstract

**Background:**

Invasions pose a large threat to native species, but the question of why some species are more invasive, and some communities more prone to invasions than others, is far from solved. Using 10 different three-species bacterial communities, we tested experimentally if the phylogenetic relationships between an invader and a resident community and the propagule pressure affect invasion probability.

**Results:**

We found that greater diversity in phylogenetic distances between the members of resident community and the invader lowered invasion success, and higher propagule pressure increased invasion success whereas phylogenetic distance had no clear effect. In the later stages of invasion, phylogenetic diversity had no effect on invasion success but community identity played a stronger role.

**Conclusions:**

Taken together, our results emphasize that invasion success does not depend only on propagule pressure, but also on the properties of the community members. Our results thus indicate that invasion is a process where both invader and residing community characters act in concert.

**Electronic supplementary material:**

The online version of this article (doi:10.1186/s12898-017-0126-z) contains supplementary material, which is available to authorized users.

## Background

Invasions of non-native species are considered a serious threat to native species across the globe. However, our ability to predict which species are invasive, and in what kind of communities invasions take place, is far from complete. Interestingly, understanding the reasons for the invasion success of certain species or populations seems to have gained more attention than those for why some communities are more prone to invasions [1, 2]. Perhaps the most renowned mechanistic explanation for invasions is the effect of propagule pressure. The amount of invading individuals, or the number of repeated introductions of invader species, have been found to positively affect the establishment of the invader, in both wild and laboratory experiments [1]. However, as the invasion process is a function of both the resident and invader species, not all communities are expected to be similar in their receptiveness to invasions. However, why some communities are more prone to invasions than others has received less attention than why some species are better invaders than others. The average and the variation in phylogenetic relatedness between the resident and invading species are likely drivers of community receptiveness to invasions.

The idea that community structure and, in particular, relatedness between an invader and the resident community could affect invasion probability has a long history [3, 4]. However, a consensus has not been reached on whether low or high phylogenetic relatedness could promote invasions [1, 5–10]. On the one hand competition-relatedness hypothesis, [9] suggests that the competitive exclusion of closely related species restricts invasion, for example because of overlapping resource use, as well as shared predators, herbivores, and pathogens. On the other hand, it has been proposed that invaders that are closely phylogenetically related to the resident species could have better invasion success due to shared mutualists, facilitation, and an increased likelihood of tolerating similar conditions [1, 11]. Such opposite hypotheses could very well explain the lack of evidence for phylogenetic relatedness affecting invasion success in the wild, where these effects cannot be controlled. However, even in controlled experiments the evidence has been mixed. For example, a study of pairwise competition with protists and rotifers found support for the hypothesis that larger phylogenetic distances promote co-existence [6]. In contrast, in pairwise comparisons of algae [7, 8] and an extensive competitive experiment with 142 species of plants [9], coexistence was not affected by phylogenetic distance. Similarly, experimental evidence using several different kinds of two, three and four-species communities gave support to the idea that close average phylogenetic distances between the invader and resident community members resulted in reduced population sizes of the invader [12]. But again, evidence obtained from recent plant studies was equivocal (see [5]).

In addition to average phylogenetic distance, the likelihood of finding a free niche space could be affected by phylogenetic diversity within community. High phylogenetic diversity could affect the invaders chance of finding a free niche space, and thus the likelihood of invasion. The very limited existing evidence suggests that in plants large phylogenetic diversity decreases the receptiveness of communities to invasion [5, 13]. This mechanism is analogous to the mechanisms believed to be operating at species diversity level where invasion is expected to be smaller in more species diverse communities. This idea has gained support from small-scale experiments of species diversity [14, 15], but not from observations on a grander scale [16–18].

We designed, and ran an invasion experiment in which highly competitive bacteria *Serratia marcescens* invaded ten different kinds of three species bacterial communities over several bacterial generations. Our experimental setup allows us to test whether invasion success is positively or negatively linked to the phylogenetic distance between the invader and the community members (estimated from 16 sRNA based phylogeny). Under positive linkage, large average phylogenetic distance should facilitate *S. marcescens* invasion, whereas the opposite would be true if small phylogenetic distance facilitated invasion success. Moreover, we can test if greater variation in phylogenetic distances between the invader and resident community members potentiates invasion success, by lessening the competition. In addition, we tested the role of propagule pressure in invasion success by manipulating the amounts of individuals starting the invasion.

## Methods

### Experimental invasions

We initiated the experiment by creating ten different three-species bacterial communities out of five species: *Pseudomonas chlororaphis (ATCC 17418), Pseudomonas putida (ATCC 12633), Escherichia coli (ATCC 11775), Enterobacter aerogenes (ATCC 13048)* and *Leclercia adecarboxylata (ATCC 23216)* (Table 1). We selected easily culturable, laboratory-adapted, species to ease identification and culturing. All community species were propagated separately from frozen samples (1:1 high-density bacteria in nutrient broth and 80% glycerol at −80 °C) for 3 days at 30 °C prior to creating the communities [40 µl of thawed bacterial stock in 4 ml of Nutrient Broth (NB: 10 g Nutrient broth, 2.5 g Yeast extract in 1 l ddH_2_O)]. The communities were initiated by pipetting 50 µl of each species (altogether 150 µl) into 6 ml of NB in 15 ml centrifuge tubes. After 3 days of propagating the communities (at 30 °C in thermally controlled cabinet; ILP-12, Jeio Tech, Seoul, Korea), we performed invasions with *S. marcescens*, using four different propagule pressures (i.e. inoculum sizes): 12.5, 25, 50 and 75 µl, respectively. We did two replicates for each propagule pressure treatment, leading to a total of eight similar communities, which were invaded with four different amounts of the invader. In total we had 80 communities. The invading *S. marcescens* had been pre-grown for 3 days using the same procedure as with the community species.

**Table 1:** List of species used in the communities (1–10) to which *S. marcescens* invaded

#	Resident species	Phylogenetic distance	Phylogenetic diversity
1	*Pseudomonas chlororaphis* + *Pseudomonas putida* + *Escherichia coli*	*0.109*	4.60E−03
2	*Pseudomonas chlororaphis* + *Pseudomonas putida* + *Enterobacter aerogenes*	*0.105*	5.59E−03
3	*Pseudomonas chlororaphis* + *Pseudomonas putida* + *Leclercia adecarboxylata*	*0.107*	5.25E−03
4	*Pseudomonas chlororaphis* + *Escherichia coli* + *Enterobacter aerogenes*	0.066	5.08E−03
5	*Pseudomonas chlororaphis* + *Escherichia coli* + *Leclercia adecarboxylata*	0.067	4.90E−03
6	*Pseudomonas chlororaphis* + *Enterobacter aerogenes* + *Leclercia adecarboxylata*	0.063	5.38E−03
7	*Pseudomonas putida* + *Escherichia coli* + *Enterobacter aerogenes*	0.066	5.16E−03
8	*Pseudomonas putida* + *Escherichia coli* + *Leclercia adecarboxylata*	0.068	4.98E−03
9	*Pseudomonas putida* + *Enterobacter aerogenes* + *Leclercia adecarboxylata*	0.064	5.47E−03
10	*Escherichia coli* + *Enterobacter aerogenes* + *Leclercia adecarboxylata*	0.024	*3.73E*−*05*

Phylogenetic distances reflect average distances between resident community species and invader and phylogenetic diversity reflects the variance of distances between the invader and resident species (see Additional file 1 for phylogeny). Italic font in phylogenetic distance indicate communities belonging to group with large phylogenetic distance. In phylogenetic diversity italic font indicate a community with very low phylogenetic diversity

On days 3 and 6 after invasion we transferred the communities to fresh medium. A well-shaken inoculum (500 µl) of each bacterial community was transferred to 5.4 ml of fresh NB medium. To mimic immigration, and to keep the community treatment effective throughout the experiment, we also added 33 µl of each pre-grown (3 days old stock) community species (according to community composition) into the communities. On 3, 6 and 9 days after invasion samples of communities were frozen (1:1 bacteria and 80% glycerol in cryotubes at −80 °C). The experiment continued at 30 °C for 9 days after the invasion.

### Invasion success

Invasion success was determined from frozen samples collected at days 3 and 9 after *S. marcescens* invasion. The population sizes of the invader and the rest of the community were determined using standard dilution series techniques (100 µl of thawed sample into 900 µl of sterile dH_2_O water. Dilution was repeated six times to achieve a 10^−6^ dilution). Diluted samples were plated on DNase test agar with methyl green (Becton and Dickinson and Company, Sparks, MD; premade at Tammer-tutkan maljat, Tampere, Finland), which allows growth of all used species, but distinguishes *S. marcescens* clones by the clear halo around colonies [19]. The plates were propagated at room temperature. 2 days after plating, both the total number of all bacterial colonies and colonies of *S. marcescens* were quantified to measure invasion success.

### Phylogenetic effects

The phylogenetic similarity among the used bacterial species was estimated from data obtained from NCBI GeneBank nucleotide sequences database using program Mega (v. 5.0). The phylogenetic tree includes the sequences FJ971882 (*E. aerogenes*), GQ856082 (*L. adecarboxylata*), NR_041980 (*S. marcescens* ssp. *marcescens*), NR_024570 (*E. coli*), AF094736 (*P. putida*), AB680102 (*P. chlororaphis*). The phylogenetic distances between species in Table 1 (invader, and resident community members) were calculated from a distance matrix based on the estimated phylogeny (see: Additional file 1). In the analyses we consider two variables. Average *phylogenetic distance* between the community members and the invader and *phylogenetic diversity,* which is the variance of phylogenetic distances between the community members and the invader (*S. marcescens* ssp. *marcescens*).

### Data analysis

Data analysis was conducted with glmer in R (Lme4), fitting generalized mixed model with a binomial error distribution and a logit link on colony counts of the invader *S. marcescens*. To control for the differences in the total colony count the amount of colonies of other species were used as a denominator (see [20]). By this way we modelled the odds of finding *S. marcescens* colonies from agar plates. We controlled also for the fact that the same community composition was used in four different propagule pressure levels and in two technical replicates by fitting community ID as a random factor in the model. Explanatory variables were propagule pressure, phylogenetic diversity and phylogenetic distance. Since phylogenetic distance was clearly either small or large (Table 1) we classified the phylogenetic distances to two groups (low < 0.09 < high). All other predictors were fitted as continuous fixed variables standardized to a mean of zero and standard deviation of one. Data analyses were done for both time points separately and testing two competing models; one with all linear effects, and one with both all linear and all quadratic effects. These two models were not simplified and their fit to the data were compared by AIC values. Since one community (number 10, Table 1) had a very low phylogenetic diversity compared to the other 9 communities the effect of phylogenetic diversity on invasion success was not fitted for this community (i.e. data were coded for this variable as missing values). Since one community (number 10, Table 1) had a very low phylogenetic diversity compared to the other 9 communities, this community has been removed from our analyses. We however provide the model with the 10th community as a Additional file 1.

## Results

**Fig. 1:**
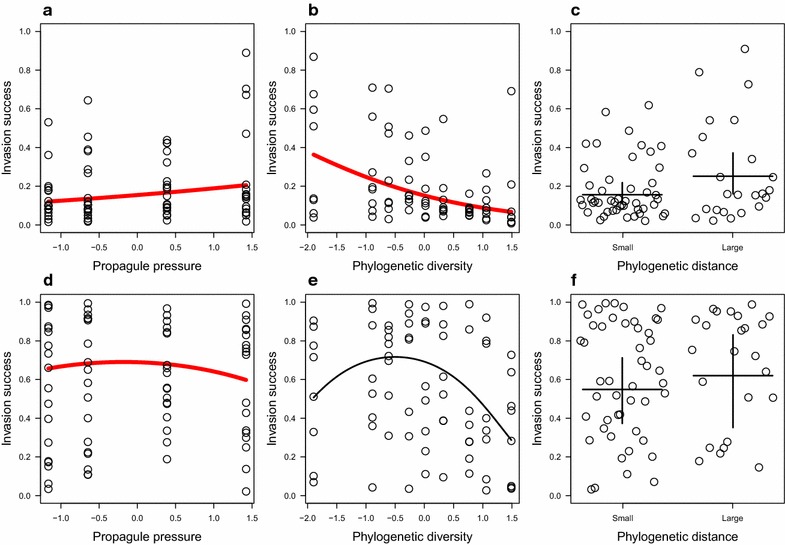
Invasion success of invader *Serratia marcescens* (proportion of invader from all colonies) three (**a**–**c**) and 9 days after the invasion (**d**–**f**). Effect of propagule pressure (**a**, **d**), phylogenetic diversity (**b**,** e**) and phylogenetic distance (**c**,** f**) on invasion success. *Panels* containing significant effects are highlighted with *red fit line* (see “Results” for detailed statistics).* Whiskers* denote ± 1.96 × standard errors of the estimate

In the beginning of the invasion (day 3), invasion success increased with increasing propagule pressure (est. = 0.245, s.e. = 0.052, z = 4.740, p < 0.001, Fig. 1a) and decreased with increasing phylogenetic diversity (est. = −0.612, s.e. = 0.167, z = −3.676, p < 0.001, Fig. 1b). In addition, large phylogenetic distance between invader and community did not favour invasion in comparison to small phylogenetic distance (small distance: 0.156, large distance: 0.252, z = 1.720, p = 0.085, Fig. 1c). Community ID had no effect on invasion (est. = 0.221, s.e. = 0.470, z = 1.484, p = 0.172). When we tested model with quadratic effects for phylogenetic diversity, and for propagule pressure, both quadratic coefficients were clearly non-significant and model fit decreased (AIC = 383.0) compared with the model with linear coefficients (AIC = 379.1, ΔAIC = 3.9).

After 9 days from invasion we found no evidence for linear effect of propagule pressure on invasion success (est. = −0.057, s.e. = 0.043, z = −1.331, p = 0.183). However, intermediate propagule pressures facilitated higher invasion success (est. = −0.158, s.e. = 0.056, z = −2.818, p = 0.005, Fig. 1d). Compared to the beginning of invasion community ID affected invasion success (est = 0.606, s.e. = 0.779, z = 2.462, p = 0.036) 9 days after invasion. No other explanatory variable affected invasion success (phylogenetic diversity: est. = −0.466, s.e. = 0.282, z = −1.651, p = 0.099; quadratic effect of phylogenetic diversity: est. = −0.463, s.e. = 0.314, z = −1.475, p = 0.140; small phylogenetic distance: 0.548, large distance: 0.620, z = 0.410, p = 0.682, Fig. 1f). The quadratic model (AIC = 588.0) with quadratic effects for phylogenetic diversity, and for propagule pressure, explained invasion better than model with only linear effects (AIC 593.8, ΔAIC = 5.8).

## Discussion

Despite the long history of invasion biology, fewer studies have focused on the properties of receiving communities which interact with the invader, than on the properties of the invaders themselves [1]. Most of the experimental studies on community properties affecting invasion have so far concentrated on exploring the effects of competition and mean phylogenetic relatedness between the invader and resident species on invasion success. However, when experiments are taken to the community level, the mean phylogenetic relatedness between species might not be the only denominator, as invasions could also be affected by phylogenetic diversity.

We found out that large phylogenetic distance was not linked strongly to invasion success (Fig. 1c). Although our results points to a direction that phylogenetically less related communities could be invaded more easily (Fig. 1c, p = 0.085), it is noteworthy that phylogenetic distance is a proxy of resource/niche use complementarity, and is sensitive to the phylogenetic history of a community [1]. For example, related species that evolved in sympatry could be less likely to compete with each other, whereas related species that evolved in allopatry could share ecological similarities, leading to strong competition (but see [21] for opposite prediction). Such effects could obscure the relation between competition and phylogenetic relatedness and perhaps be one additional reason for why evidence for the role of phylogenetic distance in invasions is mixed (see “Background”) and sometimes weak (as here). It is also evident that our dataset is relatively small (10 communities), which is suboptimal in estimating perhaps small effect of phylogenetic similarity on invasion.

In addition to phylogenetic similarity the diversity in communities could play an important role in dictating the invasion success. We found that smaller phylogenetic diversity between the invader and the invaded community promoted *S. marcescens* invasion success (Fig. 1b, e). These results could be explained if large phylogenetic diversity lowers the invader’s chances of finding a free niche space. Our results, thus, resemble those few studies done with plants, where higher phylogenetic diversity within community was found to hinder invasion [5, 13]. Moreover, our results are also in line with what has been expected to occur in invasions to species rich and poor communities.

We show that, during the early stage of invasion, the strongest explanatory variable for invasion success was the propagule pressure, as has been found in many cases before [22, 23]. Interestingly, intermediate propagule pressure at the beginning of the experiment was associated with slightly higher overall proportion of invaders at the latter stage of invasion. This finding could be linked with intrinsic population dynamics of the invader. For example those microcosms where propagule pressure has been larger could have attained maximum yield earlier than in those microcosms where propagule pressure was lower. This could lead to lowered survival after the resources have been used up (see [24]) and consequently this would cause lower invader densities, especially at latter stages of invasion when smaller differences at the beginning of the experiment would have grown larger in each renewal.

All models fitted on invasion success, measured after 9 days, indicated significant effect of community ID, whereas none of the analyses done on first time step indicated strong effects of community. This result suggest that proxies of invasion, like phylogenetic diversity could be more accurate descriptors of invasion propensity at the early stages of invasions. At latter stages individual communities could act more idiosyncratic manner, masking proxies of invasion success and emphasizing properties of particular species assemblages. We had two technical replicates, and four repeated measurements of the same community, which is relatively low sample to fully disentangle the effects of community ID on invasion success. Moreover, in fast growing bacteria, 9 days could already be considered a late stage of invasion and these results could also reflect “equilibrium” densities of these systems. Moreover, 9 days is long enough to observe evolution of invader and communities [19], which could also conceal the role of phylogenetic distance.

To summarize we found out that high propagule pressure, high phylogenetic distance and low phylogenetic diversity between the invader and the community species facilitated invasions, at early stage of invasions. At latter stage the invader numbers were more affected by the identity of the community, and to a lesser extent propagule pressure. Our results thus indicate that invasion is a process where both invader and residing community characters act in concert.

## Additional file

**Additional file 1: Figure S1.** Phylogeny of the study species, based on 16S rRNA. The tree includes the sequences FJ971882 (*E. aerogenes)*, GQ856082 (*L. adecarboxylata*), NR_041980 (*S. marcescens* ssp. *marcescens*), NR_024570 (*E. coli*), AF094736 (*P. putida*), and AB680102 (*P. chlororaphis*). The sequence accession numbers were obtained from the NCBI nucleotide sequences database. Metrics for mean phylogenetic distances and variance of distances were calculated based on standardized distances between species. Results from data analysis of full data containing all communities. **Figure S2.** Effects of propagule pressure (A), phylogenetic diversity (B) and phylogenetic distance (C) on invasion success after 3 days from invasion of depicted from quadratic model. Effects of propagule pressure (D), phylogenetic diversity (E) and phylogenetic distance (F) on invasion success after 9 days from invasion of depicted from quadratic model. Effects of propagule pressure (G), phylogenetic diversity (G) and phylogenetic distance (I) on invasion success after 9 days from invasion of depicted from model containing only linear effects (panels G-I). Both linear and quadratic models are represented due to model selection uncertainty indicated by very similar AIC values. Panels containing significant effects are highlighted with red fit line. Analysis was performed on the whole dataset. In dataset used in the paper the outlier of the phylogenetic diversity (the left most observations) were omitted from the analysis. Whiskers denote ± 1.96 × standard error of the mean.
