# Supplementary material for: Propagule pressure increase and phylogenetic diversity decrease community’s susceptibility to invasion
Source: BMC Ecol. 2017 Apr 11;17:15. doi: 10.1186/s12898-017-0126-z (PMC5387184; doi:10.1186/s12898-017-0126-z)
Supplement: Supplementary file 1 — Additional file 1: Figure S1. Phylogeny of the study species, based on 16S rRNA. The tree includes the sequences FJ971882 (E. aerogenes), GQ856082 (L. adecarboxylata), NR_041980 (S. marcescens ssp. marcescens), NR_024570 (E. coli), AF094736 (P. putida), and AB680102 (P. chlororaphis). The sequence accession numbers were obtained from the NCBI nucleotide sequences database. Metrics for mean phylogenetic distances and variance of distances were calculated based on standardized distances between species. Results from data analysis of full data containing all communities. Figure S2. Effects of propagule pressure (A), phylogenetic diversity (B) and phylogenetic distance (C) on invasion success after 3 days from invasion of depicted from quadratic model. Effects of propagule pressure (D), phylogenetic diversity (E) and phylogenetic distance (F) on invasion success after 9 days from invasion of depicted from quadratic model. Effects of propagule pressure (G), phylogenetic diversity (G) and phylogenetic distance (I) on invasion success after 9 days from invasion of depicted from model containing only linear effects (panels G-I). Both linear and quadratic models are represented due to model selection uncertainty indicated by very similar AIC values. Panels containing significant effects are highlighted with red fit line. Analysis was performed on the whole dataset. In dataset used in the paper the outlier of the phylogenetic diversity (the left most observations) were omitted from the analysis. Whiskers denote ± 1.96 × standard error of the mean. [file 12898_2017_126_MOESM1_ESM.docx]

Supplementary material: Propagule pressure increase and phylogenetic diversity decrease community’s susceptibility to invasion, by Tarmo Ketola, Kati Saarinen & Leena Lindström

**Appendix 1.**

Supplementary Figure 1.

Phylogeny of the study species, based on 16S rRNA. The tree includes the sequences FJ971882 (*E. aerogenes)*, GQ856082 (*L. adecarboxylata*), NR_041980 (*S. marcescens ssp. marcescens*), NR_024570 (*E. coli*), AF094736 (*P. putida*), and AB680102 (*P. chlororaphis*). The sequence accession numbers were obtained from the NCBI nucleotide sequences database. Metrics for mean phylogenetic distances and variance of distances were calculated based on standardized distances between species.

Analysis of full data

The raw data containing all observations from all communities indicated very similar results to tests presented in the manuscript. Only clear deviation was that instead of linear effects model (AIC=461.0) the best model explaining invasion success at the beginning of the invasion was a model including also quadratic effects (AIC=457.5, ΔAIC=3.5). This model (Figure S2a-c) confirmed the positive linear effect of propagule pressure (est.=0.269, s.e.= 0.053, z=5.102, p< 0.001, Figure 2a) in explaining the invasion success. The propagule pressure did not have quadratic effects the (est.=-0.055 s.e.=0.068, z= -0.816, p= 0.414) on invasion success. Large phylogenetic diversity (i.e. the variance of phylogenetic distances between the community members and the invader) constrained the invasion (est.=-2.793, s.e.= 0.684, z= -4.080, p<0.001). Analysis indicated also significant curvature (est.= -0.837, s.e.=0.266, z=-3.146, p=0.002, Figure S2b) on this relationship. Moreover, phylogenetic distance (i.e. phylogenetic distances between the community members and the invader) did not clearly affect invasion. If anything, the large phylogenetic distances facilitated invasion (small: 0.182, large: 0.297, z=1.957, p= 0.050, Figure S2c). Community ID did not affect invasion success (est.= 0.191, s.e. = 0.437, z=1.380, p=0.201).

Nine days after the invasion the best model with both linear and quadratic effects (AIC = 650.5), had a slightly better fit than the model with only linear effects (AIC = 651.6, ΔAIC=1.1). This rather negligible AIC difference suggests model selection uncertainty and hence both linear and quadratic models are presented below.

Nine days after the invasion the model with quadratic effects (Figure S2d-f) indicated that intermediate propagule pressures are associated with slightly better invasion success (est.=0.107, s.e.= 0.053, z=-2.010, p=0.044, Figure 2d). All other measured effects were non- significant. Linear effect of propagule pressure (est.-0.058, s.e.= 0.041, z=-1.428, p=0.1534), linear effect of phylogenetic diversity (est.=-1.570, s.e.=1.219, z=-1.288, p=0.1979), quadratic effect of phylogenetic diversity (est:=-0.483, s.e.= 0.476, z=-1.015, p=0.310) and phylogenetic distance to invader (small: 0.627 large: 0.540, z=-0.614, p=0.539) had no effects on invasion success. However, community ID explained significantly invasion success (est.=0.669, s.e.=0.818, z=2.587, p=0.029).

Model containing only linear effects (Figure S2g-i) indicated negative effect of propagule pressure on invasion success (est.=-0.092, s.e.=0.037, z=-2.457, p=0.014) nine days after the invasion. Invasion success at nine days after invasion was not affected by phylogenetic diversity (est.=-0.361, s.e.= 0.283, z=-1.277, p=0.202) or phylogenetic distance (small: 0.627, large: 0.542, z=-0.571, p=0.568). However, contrast to the beginning of the invasions community ID explained invasion success (est.=0.741, s.e.=0.861, z=2.722, p=0.024) at the latter stages of invasion.

**Supplementary Figure 2.** Effects of propagule pressure (A), phylogenetic diversity (B) and phylogenetic distance (C) on invasion success after three days from invasion of depicted from quadratic model. Effects of propagule pressure (D), phylogenetic diversity (E) and phylogenetic distance (F) on invasion success after nine days from invasion of depicted from quadratic model. Effects of propagule pressure (G), phylogenetic diversity (G) and phylogenetic distance (I) on invasion success after nine days from invasion of depicted from model containing only linear effects (panels G-I). Both linear and quadratic models are represented due to model selection uncertainty indicated by very similar AIC values. Panels containing significant effects are highlighted with red fit line. Analysis was performed on the whole dataset. In dataset used in the paper the outlier of the phylogenetic diversity (the left most observations) were omitted from the analysis. Whiskers denote ±1.96 × standard error of the mean.
